# Energy Efficient Resource Allocation for M2M Devices in LTE/LTE-A

**DOI:** 10.3390/s19245337

**Published:** 2019-12-04

**Authors:** Hajer Ben Rekhissa, Cecile Belleudy, Philippe Bessaguet

**Affiliations:** 1 LEAT / CNRS UMR 7248, University Cote Azur, 06903 Sophia Antipolis, France; cecile.belleudy@univ-cotedazur.fr; 2 iQsim, 06560 Valbonne, France; bessaguet@iqsim.com

**Keywords:** LTE/LTE-A, M2M, MTC, power saving, resource allocation, uplink scheduling

## Abstract

Machine-to-machine (M2M) communication consists of the communication between intelligent devices without human intervention. Long term evolution (LTE) and Long-term evolution-advanced (LTE-A) cellular networks technologies are excellent candidates to support M2M communication as they offer high data rates, low latencies, high capacities and more flexibility. However, M2M communication over LTE/LTE-A networks faces some challenges. One of these challenges is the management of resource radios especially on the uplink. LTE schedulers should be able to meet the needs of M2M devices, such as power management and the support of large number of devices, in addition to handling both human-to-human (H2H) and M2M communications. Motivated by the fundamental requirement of extending the battery lives of M2M devices and managing an LTE network system, including both M2M devices and H2H users, in this paper, two channel-aware scheduling algorithms on the uplink are proposed. Both of them consider the coexistence of H2H and M2M communications and aim to reduce energy consumption in M2M devices. The first algorithm, called FDPS-carrier-by-carrier modified (CBC-M), takes into account channel quality and power consumption while allocating radio resources. Our second algorithm, recursive maximum expansion modified (RME-M), offers a balance between delay requirement and energy consumption. Depending on the system requirements, RME-M considers both channel quality and system deadlines in an adjustable manner according to M2M devices needs. Simulation results show that the proposed schedulers outperform the round-robin scheduler in terms of energy efficiency and have better cell spectral efficiency.

## 1. Introduction

Today, the world is more connected than ever thanks to the machine-to-machine (M2M) communication, also called machine-type-communication (MTC). M2M describes the exchange of data between smart devices and remote servers. These intelligent machines integrate computing capabilities that enable them to capture data around them and share this with other devices without the need of human intervention. Moreover, M2M applications are used in many domains, including e-health monitoring, remote security, smart grids, smart homes, transportation systems and so on [1,2]. Cellular networks, such as 2G, 3G and long term evolution (LTE)/long-term evolution-advanced (LTE-A), play a fundamental role in the deployment of M2M communication, thanks to their ubiquitous coverage and their long range. Besides, dedicated M2M cellular architecture, as LORA and Sigfox [3] are built to provide high coverage and very low cost connectivity. However, they support only very low throughput, on the order of few bytes per minutes. Enhanced machine type communication (eMTC) and narrow band internet of things (NB-IOT) are built from existing LTE functionalities [4]. LTE/LTE-A offers high data rate, low latency, high capacity and more flexibility. However, most of the M2M devices are battery driven, and sometimes it is difficult and even impossible to replace the battery. For this reason, reducing power consumption is crucial for M2M devices. A possible solution to extend a device’s life is the use of power-aware resource allocation techniques in the uplink, as the main traffic of M2M devices is in the uplink side.

In this paper, we propose two uplink resource allocation strategies which take into account the coexistence H2H/M2M and which aim to reduce energy consumption for MTC devices. Both proposed schedulers consider channel quality while allocating radio resources for active devices with two different allocation algorithms. Moreover, the second scheduler considers the delay tolerance of MTC devices, which can vary from a few milliseconds to several hours [5], when allocating the limited resource blocks. The proposed schedulers are able to reduce the total transmission power while satisfying the quality of service (QoS) requirements in term of delay for the second scheduler.

The paper is organised as follows. Section 2 describes, briefly, the M2M communication, the packet scheduler for the LTE network and presents some works from the literature. Section 3 details system model and proposed schedulers. Section 4 discusses the results of simulation, and finally, Section 5 concludes this paper.

## 2. Overview and Related Works

In this section, we start by briefly describing the M2M communication and the packet scheduling in the LTE network, in order to provide the requisite background of this article’s proposal. Then, we will discuss some solutions proposed in the literature which study uplink scheduling for M2M in LTE/LTE-A networks.

### 2.1. M2M Communication

M2M communication describes the exchange of data between smart devices and remote servers. These intelligent machines integrate computing capabilities that enable them to capture data around them and share it with other devices without the need for human interaction. The characteristics of the M2M network are quite different from the H2H network [6,7]. The M2M network is composed of a large number of machines. According to Machina Research [8], the number of these intelligent devices will reach 27 billion in 2024. Besides, in M2M technology, the traffic is mainly in the uplink side, from the M2M device to the base station, contrarily of H2H communication, where the traffic is almost all at the downlink side. In addition, the majority of M2M devices are used to send only a few packets with minimal data.

The majority of M2M applications can be divided into two categories [5]:

- Event-driven applications: when an event occurs in the MTC device, the latter has to establish a connection with the base station, called evolved NodeB (eNB), to send messages to the server. These applications are mainly real-time applications.
- Time-driven applications: MTC devices send their data on a regular basis. The transmitting interval can vary from a few milliseconds to several minutes or hours. These applications are delay tolerant. Most M2M devices are included in this category.

### 2.2. Packet Scheduler for LTE Network

**Figure 1:**
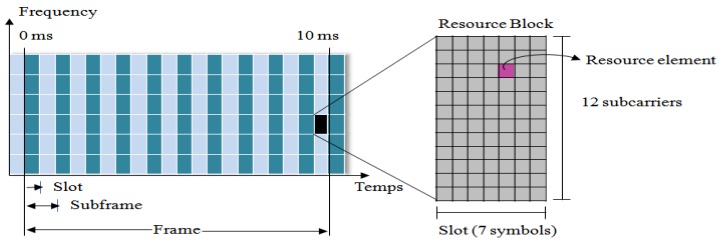
Frame in long term evolution (LTE).

LTE uses orthogonal frequency division multiplexing (OFDM) for the downlink and single carrier frequency division multiple access (SC-FDMA) in the uplink [9]. The latter has the advantage of enhancing the power efficiency of users, thanks to its low peak to average power ratio (PAPR) compared to OFDM. In LTE, both the uplink and downlink transmissions are divided into frames of duration of 10 ms, where each frame is composed of 10 sub-frames (1 ms). Each sub-frame contains two slots (0.5 ms), where each slot can contain N resource blocks (from 6 to 110 RBs) [9] depending on the bandwidth allocation. The resource element is the smallest modulation structure and consists of one symbol of 15 kHz. These resource elements are grouped to form a resource block, RB (Figure 1).

**Figure 2:**
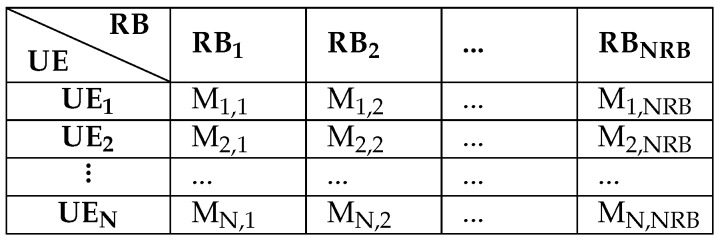
UE-RB metric matrix M.

All the allocations of RBs are handled by a scheduling function at the medium access control (MAC) layer of the eNB. When a user has data to transmit, it sends an uplink scheduling request to the base station and reports information about buffered data sizes. The scheduler in the eNB receives in input the matrix M, which has N rows corresponding to the number of active users in the cell and N_RB_ columns according to the number of RBs which can be scheduled. Each element, M_i,j_, of this matrix represents a metric value that is achieved from the utility function, where M_i,j_ denotes the metric value for user *i* and RB_j_. Figure 2 shows the UE-RB metric matrix, where UE (user equipment) designates user, which can be an H2H user or M2M device. The scheduler allocates *m* RBs to *n* users according to a defined algorithm. Each RB can be allocated only to one user and each user can be assigned to a set of contiguous RBs because of the use of SC-FDMA. The scheduler in the eNB sends the allocation map to active users over the physical downlink control channel (PDCCH). When receiving the permission to transmit, the user sends its packets as required in the allocation map which specifies the transmission time and the assigned RBs.

The LTE standard does not specify how the allocation of resource blocks is carried out. Consequently, there are many scheduling algorithms with different objectives that are proposed in the literature.

In LTE, in order to reduce complexity, most schedulers can be divided into two steps:

- Time domain packet scheduling (TDPS): In the first step, a group of devices are selected to be scheduled based on diverse metrics: delay, channel quality identifier (CQI) reports, buffer size and so forth. TDPS does not allocate resource blocks to devices.
- Frequency domain packet scheduling (FDPS): In the second step, the select users are assigned contiguous RBs using a defined algorithm.

### 2.3. Related Works

Many studies have addressed radio resource allocation for M2M devices in LTE/LTE-A technology [10,11,12,13,14,15,16,17,18,19,20,21,22,23,24,25,26,27,28,29].

In [10], a thorough survey on LTE uplink schedulers for M2M devices is presented. In this survey, authors indicate that existing schedulers could be classified into three main categories as follows: (i) power saving schedulers which aim to reduce energy consumption in M2M devices [11,12]; (ii) QoS based schedulers which aim to provide QoS handling for each type of M2M applications [13,14]; (iii) multi-hop schedulers. Multi hop communication is proposed to reduce the number of base stations and improve system performance by coverage extension [15,16].

Ghavimi et al. [17] proposed the use of group-based radio resource allocation, where M2M devices are clustered based on their transmission protocols and their QoS requirements. The algorithm aims to maximise sum-throughput while satisfying SC-FDMA constraints and QoS requirements of M2M devices.

Carlesso et al. [18] proposed an uplink LTE scheduler that considers the smart metring and real-time traffic coexistence. In this propositional scheduler, a set of relays is considered to provide the link among the base station and the smart meters. Resource blocks are allocated to users considering their QoS class identifier (QCI) and their channel quality. The authors argue that the mechanism outperforms traditional schedulers by serving more users while satisfying their delay constraint.

In [19], authors proposed a combination between round robin (RR) and first maximum expansion (FME) [30]. Depending on the type of traffic flow, either RR or FME schedulers is applied. RR is used for real time M2M applications, whereas FME is used for non-real time applications. This mechanism allows one to maximise the throughput and to ensure the fairness among users with higher priority for real time applications, but it does not consider power consumption constraints for M2M devices.

In [20], Mostafa et al. proposed a statistical priority scheduler which allocates RBs to an M2M device having the lowest statistical priority metric. This metric is a term that indicates the uniqueness of the information carried by certain data packets sent by machine type communications devices (MTCDs).

References [21,22,23,24] considered both channel condition and maximum delay tolerance when allocating resource blocks to M2M devices. Lioumpas et al. in [24] proposed two uplink scheduling schemes for the LTE based cellular systems with different objectives. The first one allocates RBs according to the device’s channel quality. However, the second algorithm prioritises devices with less delay tolerance and tries to allocate RBs with the best channel quality to that device. Both proposed schedulers do not ensure fairness between devices.

In [25,26], delay-aware uplink schedulers were proposed. Both studies classify the M2M applications into classes and give the highest priority to the critical M2M class.

Elhamy et al., in [27], proposed M2M uplink scheduling which aims at achieving a balance between satisfying system deadline and throughput maximisation. Depending on the network operating conditions and priorities, scheduling metric is adjusted; it can be channel quality based, delay based or a combination of them.

The authors of [28] proposed a predictive packet scheduling scheme for event-driven M2M applications. In a group of M2M devices, if one device sends a scheduling request (SR) to the base station, then there is a high probability that the nearby devices will also send SRs later. Therefore, in order to reduce the delay, the scheme schedules radio resources to these nearby devices before they send their SRs. The scheduler considers only event-driven applications and does not take into account other types of M2M applications. Besides, these studies [25,26,27,28] do not consider the coexistence of M2M and H2H devices.

Authors in [29] proposed a scheduling approach for M2M devices. This mechanism uses the current and past information about resource allocations, channel quality and QoS requirements to control the impact of M2M and H2H communication and to ensure fairness in resource allocation. Maia et al. [29] propose extending the nine QoS classes defined in [31] which are adapted only to H2H applications, by adding new classes for M2M communication. Their propositional scheduler consists of two phases. In the first phase, M2M devices which will be scheduled are selected according to their delay tolerance. In the second phase, available resources are divided equally between selected M2M devices. The propositional mechanism in [29] allows one to satisfy the QoS requirements and ensure fairness in resource allocations, but it does not reduce the consumed energy for M2M devices.

In this paper, we propose two mechanisms of resource allocation which take into consideration the coexistence of M2M and H2H communications, ensure reduction of energy consumption for M2M devices and maximise the satisfaction of QoS requirements.

## 3. System Model and the Novel Schedulers

### 3.1. System Model

We consider an uplink SC-FDMA system with one eNB, n active H2H devices and m active M2M modules. Every transmission time interval (TTI) of 1 ms, according to a defined algorithm, the eNB selects users among active users to be scheduled and allocates N resource blocks to them. The transmission power, as defined by the standard [32], is given in Equation (1).

(1) $$P_{TX}=\min\left(P_{max},P_{0}+10*\log_{10}\left(M\right)+\alpha *PL+\Delta_{TF}+f\right),$$

where:

$P_{max}$ is the maximum transmission power;
$P_{0}$ is the open loop path-loss-power value;
*M* is the number of RBs allocated to the user;
α is the open loop path-loss factor;
PL is the downlink path-loss measured in the user;
$\Delta_{TF}$ is a parameter related to the used modulation scheme;
*f* is a user parameter related to closed loop correlation.

### 3.2. FDPS-Carrier-By-Carrier-Modified

Our algorithm, called FDPS-carrier-by-carrier-Modified (CBC-M) [33], considers the coexistence of H2H and M2M devices. Our scheduler is divided into two steps: TDPS and FDPS. In TDPS, the scheduler prioritises the H2H devices and reserves $L_{H2H}$ resource blocks to H2H users. In FDPS, selected H2H users are scheduled using FDPS-carrier-by-carrier (CBC) scheduler, proposed by Lee et al. [34]. CBC, which is represented in Algorithm 1, allocates RBs, starting from the first RB, to the user with best metric. Once a UE has been assigned a RB, it can no longer be assigned RBs unless it satisfies the contiguity constraint. Then, the next RB is assigned to UE with maximum value UE-RB.

**Algorithm 1** FDPS-Carrier-By-Carrier
1:Let U be the set of schedulable modules;  2:Let M[i][j] be the metric of $H2H_{j}$ module at $RB_{i}$;  3:Let A[m] be RB-to-module assignment status;  4:**for** RB c = 1 to m **do**  5:    pick the best module i∈U with largest value $M_{i,c}$;  6:    Assign $RB_{c}$ to module i (i.e. A[c]←i);  7:    Let I be RBs already assigned to module *i*;  8:    **if** I = *⌀* **then**  9:        U=U−A[c−1]  10:    **end if**;  11:**end for**.


Table 1 shows an example of RBs’ allocations using a CBC scheduler. $RB_{1}$ is assigned to $UE_{2}$ which has the highest metric. Then, because $UE_{1}$ has the highest value, $RB_{2}$ is allocated to it. At $RB_{3}$, $UE_{3}$ has the highest value, so $RB_{3}$ is assigned to it. When we reach $RB_{4}$, $UE_{1}$ has the highest value, but the resource block can not be assigned to it as it violates the contiguity constraint. However, $RB_{5}$ is assigned to $UE_{3}$ as it satisfies contiguity constraint. $RB_{6}$ again belongs to $UE_{1}$ but it can not be assigned to it again for the same reason.

The rest of the resource blocks (RBs) (Equation (2)) are assigned to M2M devices using Algorithm 2:

(2) $$L_{M2M}=N_{RBs}-L_{H2H},$$

where:

- $L_{M2M}$ is the number of RBs assigned to M2M devices;
- $N_{RBs}$ is the total resources blocks available for scheduling;
- $L_{H2H}$ designs the number of RBs assigned to H2H devices.

CBC-M starts allocating RBs to M2M devices from the first un-allocated RB. For each RB, the M2M device with the best CQI is selected. The resource block is allocated to M2M device only if the maximum power is not yet reached.

**Algorithm 2** FDPS-carrier-by-carrier-Modified
1:Let M be the set of schedulable M2M modules;  2:Let MCQI[i][j] be the metric of $M2M_{j}$ module at $RB_{i}$;  3:Let $L_{M2M}$ be the number of RBs not allocated to H2H users;  4:Let $P_{T}\left(j\right)$ be the transmission power of $M2M_{j}$;  5:**for** RB c = m+1 to $N_{RBs}$ **do**  6:    Find $M2M_{k}$ with best MCQI[c][k];  7:    **if** ($P_{T}\left(k\right)$ < $P_{max}$) **then**  8:        Assign $RB_{c}$ to $M2M_{k}$;  9:        Let I be the number of RBs already assigned to $M2M_{k}$;  10:    **end if**;  11:**end for**.


By allocating RBs with good channel quality, M2M device reduces the number of necessary RBs to send data and consequently the power transmission is reduced. However, CBC-M does not take into consideration delay constraint. For this reason, our second proposed algorithm considers power consumption and delay constraints while allocating resource blocks to M2M devices.

### 3.3. Recursive Maximum Expansion Modified

Our second algorithm, called recursive maximum expansion modified [33], takes into account the coexistence of H2H and M2M devices. It uses recursive maximum expansion (RME) scheduler [30] to assign resource blocks to H2H devices. $L_{H2H}$ RBs are reserved to H2H devices and the rest of the RBs are assigned to M2M devices. RME consists of finding the UE-RB that has the highest metric value and assigning this RB to the selected UE. Then, it expands the allocation at the right and left hand side of the RB selected for the same UE. This continues until another UE with better metric is found. A new search for highest UE-RB is done.

Table 2 shows an example of resource blocks allocation using RME. $UE_{1}$ has the highest metric at $RB_{2}$, so this latter is allocated to $UE_{1}$. Then, RME checks at the right and left side of $RB_{2}$ if $UE_{1}$ has higher values than other H2H devices. But because none of the RBs belong to it, RME starts a new search. $UE_{1}$ can no longer be assigned RBs until all UEs have been assigned an RB. The next highest value belongs to $UE_{3}$ at $RB_{5}$, so $RB_{5}$ is allocated to $UE_{3}$. At the right and left side, none of RBs belong to $UE_{3}$, so a new search is started and $RB_{3}$ is allocated to $UE_{2}$ which has the highest metric at $RB_{3}$. Lastly, the remaining RBs of $L_{H2H}$ that have not been allocated are assigned to UEs which satisfy the contiguity constraint.

**Figure 3:**
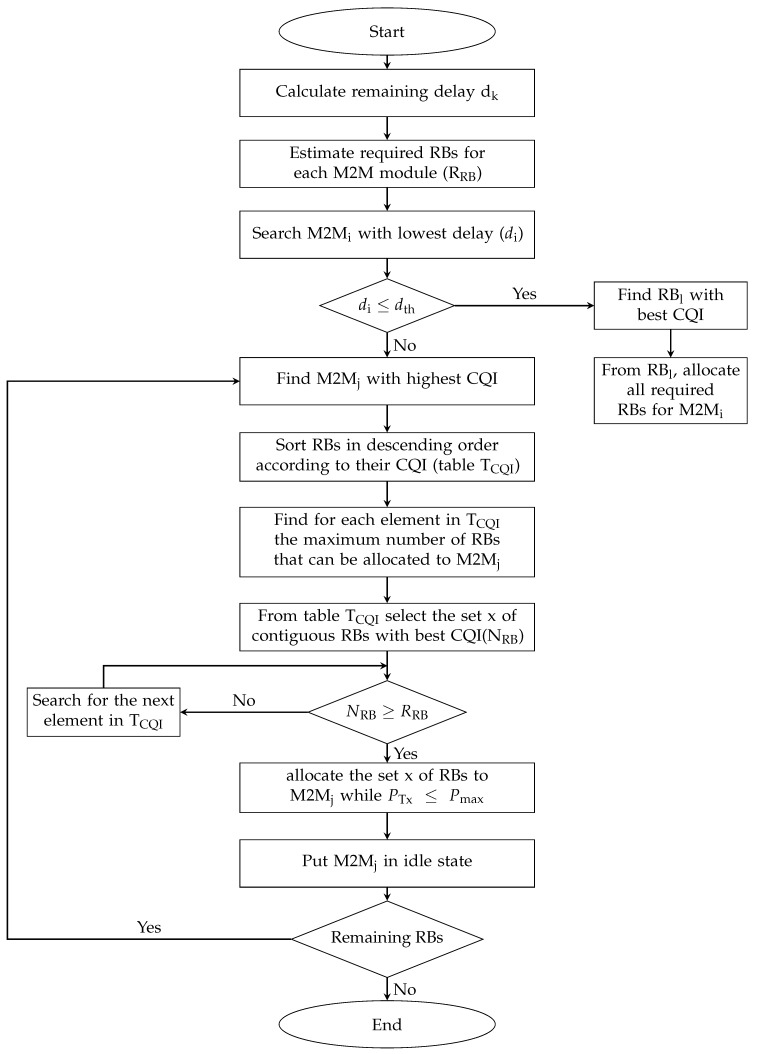
Flow chart of RME-M uplink scheduler for M2M devices.

Remaining resource blocks, $L_{M2M}$, are allocated to M2M devices using the RME-M scheduler, whose framework is shown in Figure 3. The steps of this algorithm are as follows.

Step 1. Every TTI, calculate, using RME-M, the remaining delay d_k_ which corresponds to the difference between the maximum tolerable delay for each application and time spent in the buffer.
Step 2. Sort M2M devices in ascending order according to their delay d_k_.
Step 3. If d_i_ of the i^th^ M2M device is lower than a delay threshold (d_Th_), then find the $RB_{l}$ corresponding to the best CQI. From RB_l_, allocate all the required RBs for M2M_i_. Exclude the allocated RBs from $L_{RB}^{M2M}$. Delay threshold changes dynamically according to the minimum tolerable delay for each application. It has to be higher than the tolerable delay and higher than 4 ms (time necessary for each application to prepare its data to be sent after receiving allocation table from the eNB).
Step 4. If d_i_ is higher than the delay threshold (d_th_), find M2M_j_ with the highest CQI.
Step 5. For M2M_j_, sort RBs in descending order according to their CQI (called table T_CQI_).
Step 6. Find for each element in T_CQI_, the maximum number of RBs that can be allocated to M2M_j_.
Step 7. From T_CQI_, find the first RB_k_ that allows for sending the maximum data while power transmission is lower than the maximum transmission power.
Step 8. Allocate the RB_k_ to M2M_j_.
Step 9. Expand the allocation both on the right and left hand side of RB_k_ until another M2M device has a better metric.
Step 10. Put M2M_j_ in the idle state.
Step 11. Repeat Steps 3–10 by searching for the maximum among non idled M2M devices. Stop when all RBs are allocated or all M2M devices are in the idle state.

**Figure 4:**
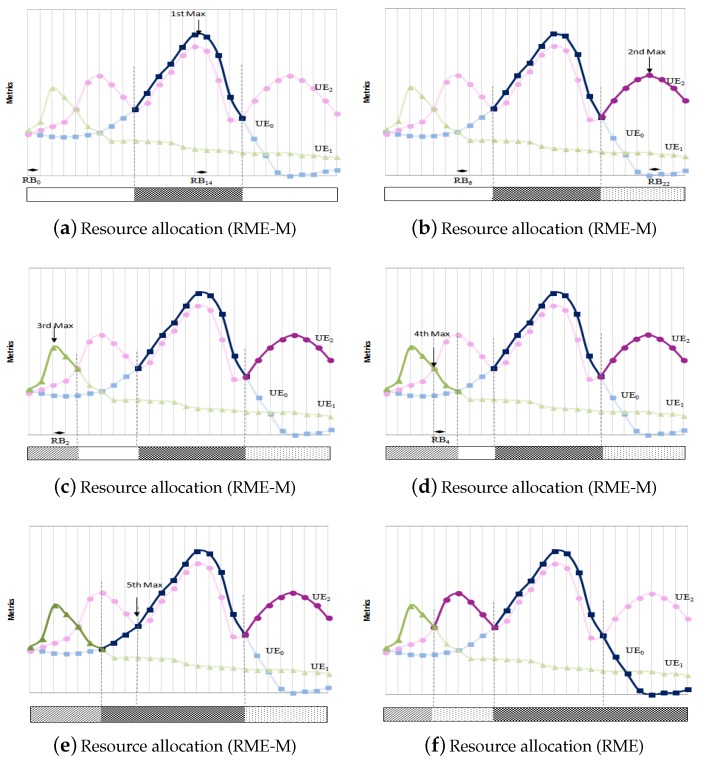
Example of resource allocation by RME-M and comparison with RME.

Figure 4 represents an example of resource blocks allocation using RME-M scheduler, if there is no user with critical delay. Figure 4a shows that $UE_{0}$ has the highest metric value at $RB_{14}$, so $UE_{0}$ is selected to be scheduled. Then a search of other higher values for $UE_{0}$ is done. In this example, $UE_{0}$ has only one maximum that allows to send data with good channel quality. So $RB_{14}$ is assigned to $UE_{0}$. RBs allocation is expanded at the right and left hand side of $RB_{14}$, until another UE with a better metric is found ($UE_{2}$). Consequently, RBs from $RB_{9}$ to $RB_{18}$ are allocated to $UE_{0}$; then, the latter is put into an idle state. The search of the next maximum among the non-idled UEs (Figure 4b) shows that $UE_{2}$ has two maximums at $RB_{6}$ and $RB_{22}$; however, the allocation of $RB_{22}$ to $UE_{2}$ allows for assigning more RBs with good channel quality than if $RB_{6}$ is allocated to $UE_{2}$. Consequently, resource blocks from $RB_{19}$ to $RB_{26}$ are assigned to $UE_{2}$. The search of the maximum among non idled UEs is repeated (Figure 4c). When all UEs are idled and not all RBs have been allocated, remaining resources are allocated to the adjacent UE with highest metric (Figure 4d,e). A comparison between the final resource blocs allocation using RME-M and RME schedulers is shown in Figure 4e,f respectively. RME-M allocates the set of RBs with best CQI, contrarily to RME (Figure 4f which only searches for the combination $UE_{i}-RB_{j}$ with the highest metric value and assigns $RB_{j}$ to $UE_{i}$ without considering metric value of adjacent RBs.

## 4. Results and Analysis

### 4.1. Simulation Environment

We used a round-robin (RR) scheduler, and CBC and RME schedulers which are channel aware schedulers, as references to evaluate our algorithms. The RR scheduler, which is one of the classic schedulers used in many older systems, consists of distributing RBs to all UEs that require data transmission equally. It assigns resource blocks to users in a circular order.

In order to evaluate the performance of our algorithms, we used the LTE-Sim simulator [35]. In our simulation, we considered a single cell, in which one eNB was positioned in the centre, whereas M2M and H2H devices were distributed randomly. In this study, voip, video and constant bit rate (CBR) flows were used for H2H communication; 30 H2H devices were used, 10 for each flow. As mentioned in Section 2.1, the majority of M2M applications are either event-driven or time-driven applications, so in this study, these two types of M2M applications were simulated. The distribution of the M2M devices was considered in our scenario as follows: 70% of devices were time-driven applications and 30% of MTC devices were event-driven applications. The burst transmission of M2M event-driven applications was modelled with a Poisson process with rate λ = 50 ms. However, the transmission interval of time-driven application was fixed and it was selected randomly within the range $\left[0.05,5\right]$ s. Both M2M applications had a packet size of 125 bytes [36]. Table 3 shows the simulation parameters and Table 4 summarises the information about the traffic model.

Besides, for RME-M scheduler, the delay threshold was fixed to one frame (10 ms), which is higher than the minimum tolerable delay of M2M applications.

### 4.2. Evaluation Metrics

In this paper, we consider the following metrics which evaluate the performance of the schedulers:

- Power efficiency: we calculated it in terms of dBm per bytes, where the power in dBm was calculated using Equation (1).
- Spectral efficiency: it is defined as the ratio between the information rate (bit/s) and the bandwidth of the channel (Hz).
- Number of packets that do not satisfy the constraint of maximum tolerable delay.
- Fairness: it determines whether users are receiving fair shares of RBs. It is measured using Jain’s fairness index [39], as shown in Equation (3).
  (3) $$J\left(T\right)=\frac{{\left(\sum_{n=1}^{N}T\left(n\right)\right)}^{2}}{N\sum_{n=1}^{N}T{\left(n\right)}^{2}},$$
  where *N* is the number of users and T(n) is the normalised throughput (Kbps) of *n*th user.

### 4.3. Performance Evaluation

In this section, we compare the performance of our proposed schedulers to the RME, CBC and RR schedulers. To ensure the minimum of fairness between H2H and M2M devices, both RME-M and CBC-M allocated maximum 60% of the total RBs to H2H modules [13]. The rest of RBs were assigned to M2M devices.

**Figure 5:**
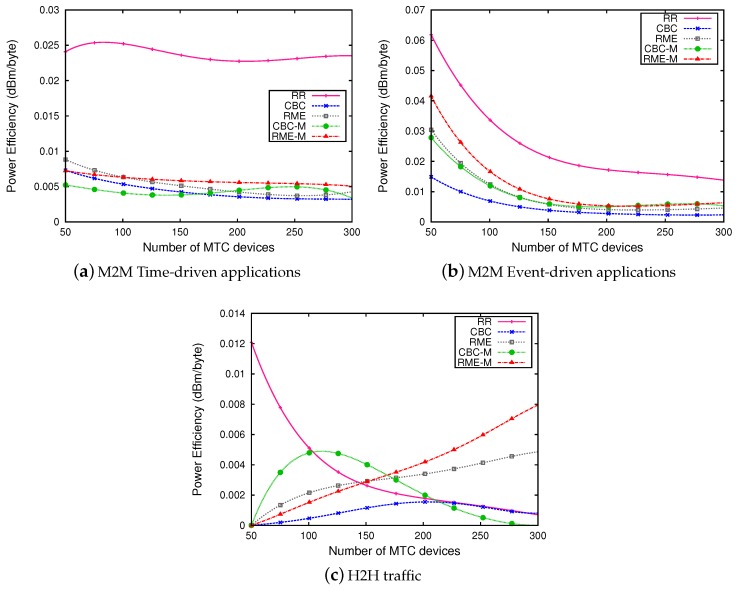
Power efficiency (dBm/byte).

Figure 5 represents the power efficiency in dBm/bytes. It shows that our proposed schedulers, RME and CBC, allow one to increase the power efficiency for both types of M2M applications compared to the RR scheduler (Figure 5a,b). This efficiency is due to the reduction of power transmission. These schedulers allow one to allocate RBs with best channel quality which has the consequence of using fewer RBs to send data, contrarily to the RR scheduler which does not consider channel quality when allocating resource blocks. Equation (1) shows that the transmitted power is proportional to the number of RBs. So if the number of allocated RBs reduces from N RBs to M RBs, the power can be saved by a value of $\Delta P_{M,N}$ (Equation (4)).

(4) $$\Delta P_{M,N}=P_{N}-P_{M}=10*\log_{10}\left(N/M\right).$$

**Figure 6:**
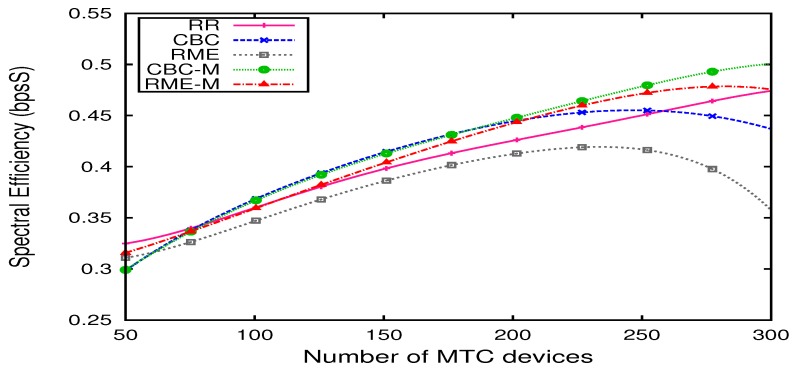
Spectral efficiency X number of M2M devices.

Figure 6 represents the spectral efficiencies of RR, CBC, RME and our proposed schedulers. We note that our schedulers enable one to have better spectral efficiency than RR scheduler, thanks to the allocation of RBs with the best CQI. Basilashvili [40] demonstrated that spectral efficiency, which is the measure of wireless network capacity, is proportional to channel quality. Using the Shannon–Hartley theorem (Shannon’s Law), the channel capacity is:

(5) $$C\approx n*B*\log_{2}S/N,$$

where C is the channel capacity (bits/s), n represents the number of transmit antennaes, B is the bandwidth and S/N is the signal-to-noise ratio. This theorem defines the maximum rate at which information can be transmitted over a communication channel, over the air interface in our case, of a specified bandwidth in the presence of noise. Besides, from Figure 6, we noticed that RME-M has better spectral efficiency than RME scheduler thanks to the allocation of a set of RBs with best channel quality.

**Figure 7:**
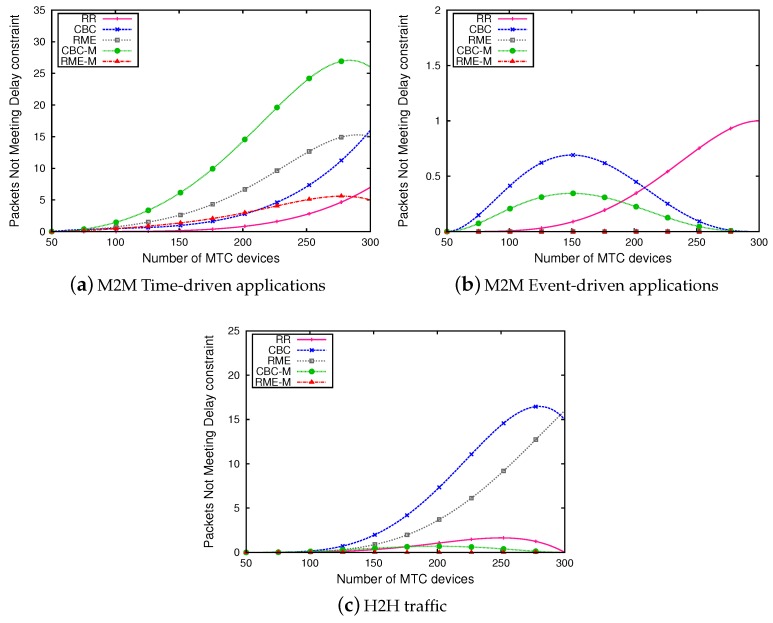
Number of packets not meeting delay constraint.

Figure 7 represents the number of packets not satisfying delay constraint. We noticed that RME-M allows one to reduce, considerably, the number of packets not meeting their delay constraint. As in addition to taking into consideration power consumption, RME-M takes into account the delay constraint, which allows devices with less delay tolerance to have the highest priority and send their data before their delay expiration. Contrarily to CBC-M scheduler, which does not consider delay constraint, RME-M performs very well and achieves a very high percentage of MTC devices served.

**Figure 8:**
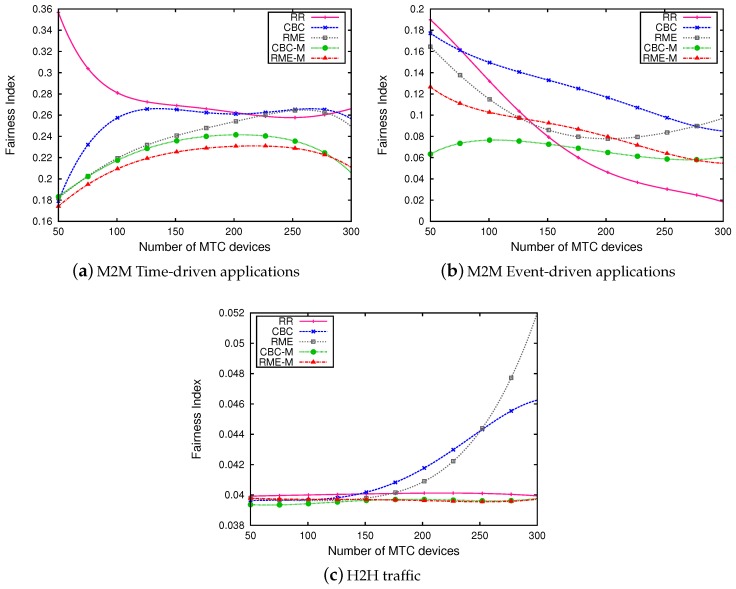
Fairness index.

Figure 8 represents the results in term of fairness. The results show that RR scheduler has the best fairness for M2M periodic devices (Figure 8a). However, all other schedulers have a similar fairness index; this is due to the fact that RR scheduler equally allocates RBs to all devices without considering their channel quality. Our schedulers allow similar fairness for event driven applications (Figure 8b). For H2H devices, RME and CBC have better fairness indexes than our propositional schedulers. This is due the limitation of the number of resource blocs allocated to H2H devices imposed by our proposed schedulers. In CBC and RME, H2H devices are prioritised and if they have better channel quality than M2M devices, the latter will suffer starvation of allocated RBs.

## 5. Conclusions

In this paper, we introduced two uplink scheduling algorithms for M2M devices over LTE. Both of them take into consideration the channel quality when allocating resource blocks to devices, which has the advantage of reducing the number of RBs necessary to send data, and consequently, reducing the energy consumed by devices. CBC-M can achieve the desired objective, which is reducing energy consumption comparing to RR scheduler; however, it cannot guarantee meeting the delay requirements of M2M devices. It was also shown that RME-M, which considers delay constraint, allows one to satisfy the QoS requirements of a large number of MTC devices in terms of delay and reducing power consumption. In this paper, we have also demonstrated the superiority of our approaches, in terms of spectral efficiency.

## Figures and Tables

**Table 1:** Example with frequency domain packet scheduling (FDPS)-carrier-by-carrier.

	$RB_{1}$	$RB_{2}$	$RB_{3}$	$RB_{4}$	$RB_{5}$	$RB_{6}$
$UE_{1}$	0.52	**1.8**	0.2	1.7	0.9	0.7
$UE_{2}$	**0.9**	0.5	1	0.6	0.52	0.65
$UE_{3}$	0.71	0.4	**1.8**	**0.23**	**1.2**	0.55

**Table 2:** Example with recursive maximum expansion.

	$RB_{1}$	$RB_{2}$	$RB_{3}$	$RB_{4}$	$RB_{5}$	$RB_{6}$
$UE_{1}$	0.52	**1.8**	0.2	1.7	1.8	1.6
$UE_{2}$	0.9	0.5	**1**	**0.6**	0.52	0.65
$UE_{3}$	0.71	0.4	0.8	0.23	**1.2**	**0.55**

**Table 3:** Simulation parameters.

Parameter	Value
Bandwidth	5 MHz (25 RBs are available per TTI)
Simulation time	5 s or 5000 TTIs executed 10 times
Number of eNode B	1 with radius 1km
Mobility	3 km/h for H2H and fixed for M2M
$P_{max}$, $P_{0}$	23 dBm, −57 dBm/PRB
$\Delta_{TF}$, *f* and α	Not considered (set to zero)

**Table 4:** Traffic model.

Application Type	Number of Devices	Model	Maximum delay
Constant Bit Rate (CBR)[37]	10	128 kbps with packet size of 256 bytes	300 ms
VoIP	10	G.795 [37]	100 ms
Video	10	H264 with 128 kbps [38]	150 ms
M2M dime-driven	70%	Inter-arrival time of $\left[0.05,5\right]$ s with packet size of 125 bytes	Equal to inter-arrival time
M2M event-driven	30%	Poisson process with λ=50 ms and with packet size of 125 bytes	50 ms

## References

[1] Chen M., Wan J., Li F. (2012). Machine-to-Machine Communications: Architectures, Standards and Applications. KSII Trans. Internet Inf. Syst..

[2] Kim J., Lee J., Kim J., Yun J. (2014). M2M Service Platforms: Survey, Issues, and Enabling Technologies. IEEE Commun. Surv. Tutor..

[3] Augustin A., Yi J., Clausen T., Townsley W.M. (2016). A Study of LoRa: Long Range & Low Power Networks for the Internet of Things. Sensors.

[4] El Soussi M., Zand P., Pasveer F., Dolmans G. Evaluating the Performance of eMTC and NB-IoT for Smart City Applications. Proceedings of the IEEE International Conference on Communications (ICC).

[5] Nikaein N., Laner M., Zhou K., Svoboda P., Drajic D., Popovic M., Krco S. Simple Traffic Modeling Framework for Machine Type Communication. Proceedings of the International Symposium on Wireless Communication Systems.

[6] 3GPP (2017). Service requirements for Machine-Type Communications (MTC).

[7] ETSI TS 102 689 V2.1.1 (2013). Machine-to-Machine communications (M2M); M2M Service Requirements.

[8] Ranken M. (2015). M2M Global Forecast & Analysis 2014-24.

[9] 3GPP (2016). Physical Layer Procedures.

[10] Mehaseb M.A., Gadallah Y., Elhamy A., Elhennawy H. (2016). Classification of LTE Uplink Scheduling Techniques: An M2M Perspective. IEEE Commun. Surv. Tutor..

[11] Ghandour F., Frikha M., Tabbane S. A Fair and Power Saving Uplink Scheduling Scheme for 3GPP LTE Systems. Proceedings of the International Conference on the Network of the Future (NOF).

[12] Jeong Y., Kim M., Chung M.Y., Lee T., Choo H. (2013). Frequency-Domain Packet Scheduling for Low PAPR in 3GPP LTE Uplink. Int. J. Smart Homev..

[13] Abdalla I., Venkatesan S. A QoE preserving M2M-aware hybrid scheduler for LTE uplink. Proceedings of the International Conference on Selected Topics in Mobile and Wireless Networking (MoWNeT).

[14] Jang H., Lee Y. (2015). QoS-constrained Resource Allocation Scheduling for LTE network. Int. Symp. Wirel. Pervasive Comput. (ISWPC).

[15] Bulakci Ö., BouSaleh A., Ren Z., Redana S., Raaf B., Hämäläinen J. Two-step Resource Sharing and Uplink Power Control Optimization in LTE-Advanced Relay Networks. Proceedings of the IEEE Multi-Carrier Systems & Solutions (MC-SS).

[16] Qianrui L., Lusheng W., Cottatellucci L., Nikaein N. Low Complexity Grouping for Massive Scheduling in 4G Networks. Proceedings of the 10th International Symposium on Modeling and Optimization in Mobile, Ad Hoc and Wireless Networks (WiOpt).

[17] Ghavimi F., Lu Y.W., Chen H.H. (2017). Uplink scheduling and power allocation for M2M communications in SC-FDMA-based LTE-A networks with QoS guarantees. IEEE Trans. Veh. Technol..

[18] Carlesso M., Antonopoulos A., Granelli F., Verikoukis C. Uplink scheduling for smart metering and real-time traffic coexistence in LTE networks. Proceedings of the IEEE International Conference on Communications (ICC).

[19] Ouaissa M., Rhattoy A. (2019). QoS hybrid uplink scheduler based on service type for M2M communications in LTE networks. Indones. J. Electr. Eng. Comput. Sci..

[20] Mostafa A.E., Gadallah Y. (2017). A Statistical Priority-Based Scheduling Metric for M2M Communications in LTE Networks. IEEE Access.

[21] Wiriaatmadja D.T., Choi K.W. (2015). Hybrid random access and data transmission protocol for machine-to-machine communications in cellular networks. IEEE Trans. Wirel. Commun..

[22] Oh C.Y., Hwang D., Lee T.J. (2015). Joint access control and resource allocation for concurrent and massive access of M2M devices. IEEE Trans. Wirel. Commun..

[23] Gotsis A.G., Lioumpas A.S., Alexiou A. (2013). Analytical modelling and performance evaluation of realistic time-controlled M2M scheduling over LTE cellular networks. Eur. Trans. Telecommun..

[24] Lioumpas A.S., Alexiou A. Uplink scheduling for Machine-to-Machine communications in LTE-based cellular systems. Proceedings of the IEEE Globecom workshops.

[25] Gadallah Y., Ahmed M.H., Elalamy E. (2017). Dynamic LTE resource reservation for critical M2M deployments. Pervasive Mob. Comput..

[26] Li N., Cao C., Wang C. (2017). Dynamic resource allocation and access class barring scheme for delay-sensitive devices in machine to machine (M2M) communications. Sensors.

[27] Elhamy A., Gadallah Y. Bat: A balanced alternating technique for M2M uplink scheduling over LTE. Proceedings of the IEEE Vehicular Technology Conference (VTC Spring).

[28] Brown J., Khan J.Y. (2015). A predictive resource allocation algorithm in the lte uplink for event based M2M applications. IEEE Trans. Mob. Comput..

[29] Maia A.M., Vieira D., Castro M.F., Ghamri-Doudane Y. A mechanism for uplink packet scheduler in LTE network in the context of machine-to-machine communicatio. Proceedings of the IEEE Global Communications Conference.

[30] Temino L.R., Berardinelli G., Frattasi S., Mogensen P. Channel-aware scheduling algorithms for SC-FDMA in LTE uplink. Proceedings of the International Symposium on Personal, Indoor and Mobile Radio Communications.

[31] 3GPP (2013). Policy and Charging Control Architecture.

[32] 3GPP (2016). Evolved Universal Terrestrial Radio Access (E-UTRA); User Equipment (UE) Radio Transmission and Reception.

[33] Ben Rekhissa H., Belleudy C., Bessaguet P. Power Efficient Packet Scheduling for M2M Devices over LTE/LTE-A Technologies. Proceedings of the IEEE International Workshop on Computer Aided Modeling and Design of Communication Links and Networks (CAMAD).

[34] Lee S.B., Pefkianakis I., Meyerson A., Xu S., Lu S. Proportional Fair Frequency-Domain Packet Scheduling for 3GPP LTE Uplink. Proceedings of the IEEE INFOCOM.

[35] Piro G., Grieco L.A., Boggia G., Capozzi F., Camarda P. (2011). Simulating LTE Cellular Systems: An Open-Source Framework. IEEE Trans. Veh. Technol..

[36] 3GPP (2012). Analysis on Traffic Model and Characteristics for mtc and Text Proposal.

[37] Salah M., Ali N.A., Taha A.E., Hassanein A.E. Evaluating Uplink Schedulers in LTE in Mixed Traffic Environments. Proceedings of the IEEE International Conference on Communications (ICC).

[38] Pötsch T., Marwat S.N.K.K., Zaki Y., Gorg C. Influence of future M2M communication on the LTE system. Proceedings of the IFIP Wireless and Mobile Networking Conference (WMNC).

[39] Jain R., Chiu D., Hawe W. (1984). A Quantitative measure of fairness and discrimination for resource allocation in Shared Computer Systems. ACM Trans. Comput. Syst..

[40] Basilashvili G. (2017). Study of Spectral Efficiency for LTE Network. Am. Sci. Res. J. Eng. Technol. Sci..

